# A prospective, open-label, multicenter, observational study to evaluate the efficacy and safety of bortezomib-melphalan-prednisone as initial treatment for autologous stem cell transplantation-ineligible patients with multiple myeloma

**DOI:** 10.18632/oncotarget.16790

**Published:** 2017-04-03

**Authors:** Min Kyoung Kim, Kihyun Kim, Chang-Ki Min, Jae-Yong Kwak, Sang-Byung Bae, Sung-Soo Yoon, Je-Jung Lee, Ki Hwan Kim, Seung-Hyun Nam, Yeung-Chul Mun, Hyo Jung Kim, Sung Hwa Bae, Ho-Jin Shin, Jung-Hee Lee, Joon Seong Park, Seong Hyun Jeong, Mark Hong Lee, Yang-Soo Kim, Ho Sup Lee, Keon Woo Park, Won-Sik Lee, Sang Min Lee, Jeong-Ok Lee, Myung Soo Hyun, Deog Yeon Jo, Sung-Nam Lim, Jae Hoon Lee, Do-Yeun Cho, Young Rok Do, Jeong-A Kim, Seong Kyu Park, Jin Seok Kim, Soo-Jeong Kim, Hawk Kim, Hyeon Gyu Yi, Joon Ho Moon, Chul Won Choi, Sung-Hyun Kim, Young-Don Joo, Hoon-Gu Kim, Byung Soo Kim, Moo-Rim Park, Moo-Kon Song, Su-Youn Kim

**Affiliations:** ^1^ Department of Medicine, Yeungnam University College of Medicine, Daegu, Korea; ^2^ Department of Medicine, Samsung Medical Center, Sungkyunkwan University School of Medicine, Seoul, Korea; ^3^ Division of Hematology, Department of Internal Medicine, Seoul St. Mary's Hospital, The Catholic University of Korea, Seoul, Korea; ^4^ Department of Internal Medicine, Chonbuk National University Medical School, Jeonju, Korea; ^5^ Department of Internal Medicine, Soonchunhyang University Hospital, Cheonan, Korea; ^6^ Department of Internal Medicine, Seoul National University Hospital, Seoul, Korea; ^7^ Department of Hematology/Oncology, Chonnam National University Hwasun Hospital, Hwasun, Korea; ^8^ Department of Internal Medicine, Seoul National University Boramae Medical Center, Seoul, Korea; ^9^ Department of Internal Medicine, VHS Medical Center, Seoul, Korea; ^10^ Department of Internal Medicine, Ewha Womans University School of Medicine, Seoul, Korea; ^11^ Department of Internal Medicine, Hallym University Sacred Heart Hospital, Hallym University College of Medicine, Anyang, Korea; ^12^ Department of Internal Medicine, Catholic University of Daegu School of Medicine, Daegu, Korea; ^13^ Department of Internal Medicine, Division of Hematology/Oncology, School of Medicine, Medical Research Institute, Pusan National University Hospital, Busan, Korea; ^14^ Department of Hematology, Asan Medical Center, University of Ulsan College of Medicine, Seoul, Korea; ^15^ Department of Hematology/Oncology, Ajou University School of Medicine, Suwon, Korea; ^16^ Department of Internal Medicine, Konkuk University Medical Center, Seoul, Korea; ^17^ Department of Hematology/Oncology, Kosin University College of Medicine, Busan, Korea; ^18^ Department of Internal Medicine, Dankook University College of Medicine, Cheonan, Korea; ^19^ Department of Internal Medicine, Inje University Busan Paik Hospital, Busan, Korea; ^20^ Department of Internal Medicine, Seoul National University Bundang Hospital, Gyeonggi-do, Korea; ^21^ Department of Hematology/Oncology, Chungnam National University Hospital, Daejeon, Korea; ^22^ Department of Internal Medicine, Haeundae Paik Hospital, Inje University College of Medicine, Busan, Korea; ^23^ Department of Internal Medicine, Gachon University Gil Medical Center, Incheon, Korea; ^24^ Department of Internal Medicine, Konyang University Hospital, Daejeon, Korea; ^25^ Department of Internal Medicine, Keimyung University School of Medicine, Daegu, Korea; ^26^ Department of Hematology, College of Medicine, The Catholic University of Korea, Seoul, Korea; ^27^ Department of Internal Medicine, Soonchunhyang University Bucheon Hospital, Bucheon, Korea; ^28^ Division of Hematology, Department of Internal Medicine, Severance Hospital, Yonsei University College of Medicine, Seoul, Korea; ^29^ Division of Hematology and Cellular Therapy, Ulsan University Hospital, University of Ulsan College of Medicine, Ulsan, Korea; ^30^ Department of Internal Medicine, Inha University Hospital, Inha University School of Medicine, Incheon, Korea; ^31^ Department of Hematology/Oncology, Kyungpook National University Hospital, Daegu, Korea; ^32^ Department of Internal Medicine, Korea University Guro Hospital, Korea University College of Medicine, Seoul, Korea; ^33^ Department of Internal Medicine, Dong-A Medical Center, Dong-A University College of Medicine, Busan, Korea; ^34^ Department of Hemato-Oncology, Inje University Haeundae Paik Hospital, Busan, Korea; ^35^ Department of Internal Medicine, Gyeongsang Institute of Health Sciences, Gyeongsang National University School of Medicine and Gyeongsang National University Changwon Hospital, Changwon, Korea; ^36^ Department of Hematology, Korea University Medical Center, Korea University College of Medicine, Seoul, Korea; ^37^ Department of Internal Medicine, School of Medicine, Wonkwang University, Iksan, Korea; ^38^ Department of Hemato-Oncology, Hanyang University Hanmaeum Changwon Hospital, Changwon, Korea; ^39^ Medical Affairs, Janssen Korea, Seoul, Korea

**Keywords:** multiple myeloma, aged, bortezomib, drug therapy, combination

## Abstract

Bortezomib-melphalan-prednisone (VMP) showed superior efficacy versus MP as first-line treatment for transplantation-ineligible multiple myeloma (MM). This study investigated the efficacy of VMP for Korean patients with MM.

Overall, 177 MM patients received 9 cycles of VMP in this prospective, multicenter, observational study. The primary endpoint was 2-year progression-free survival (PFS).

Thirty-nine (22%) patients were aged ≥ 75 years and 83 (47.4%) patients had International Staging System stage III. A median of 5 cycles were delivered. Overall response rate (ORR) was 72.9%, and complete response (CR) rate was 20.3%. With a median follow-up of 11.9 months, median PFS was 17 months. The 2-year PFS and overall survival (OS) rates were 29.2% and 80.0%, respectively. Median OS was not reached. PFS was significantly different depending on performance status (Eastern Cooperative Oncology Group < 2 vs. ≥ 2; *p* = 0.0002), β_2_-microglobulin level (< 5.5 vs. ≥ 5.5 mg/L; *p* = 0.0481), and cumulative dose of bortezomib (< 35.1 vs. ≥ 35.1 mg/m^2^; *p* < 0001). The common adverse events (AEs) were in line with the well-known toxicity profiles associated with VMP.

In conclusion, VMP is a feasible and effective front-line treatment for transplant-ineligible older patients with MM in Korea. Continuing therapy with prompt adjustment of treatment according to AEs may be important to improve outcomes of elderly patients.

## INTRODUCTION

Multiple myeloma (MM) is a progressive plasma cell neoplasm characterized by reduced resistance against infection, skeletal injuries (bone pain and fracture), renal failure, and anemia. The prognosis is mostly recurrent, with a median survival of approximately 3 to 4 years. Melphalan-prednisone (MP) has been the standard therapy for patients with newly diagnosed MM for over 40 years, and is associated with a median survival of 29 to 37 months [1, 2]. Studies have shown that treatment with MP resulted in partial response (PR) or greater responses in approximately half of the treated patients; however, complete response (CR) was relatively rare and median survival was approximately 3 years [3–6]. During the past decade, high-dose therapy with hematopoietic stem cell transplantation has become the preferred treatment for patients under the age of 65 years, but older patients and patients with clinically significant comorbidities usually do not tolerate this treatment.

With increasing incidence of MM among patients aged ≥ 70 years, it is becoming increasingly important to investigate treatment options in patients who are not eligible for autologous stem cell transplantation [7]. Several recent studies have proved the efficacy and safety of bortezomib in patients with recurrent or refractory MM [8] and the Assessment of Proteasome inhibition for Extending Remissions (APEX) study has demonstrated the efficacy and safety of bortezomib as secondary therapy [9]. Based on the results of the Velcade as Initial Standard Therapy in Multiple Myeloma (VISTA) study, which showed a significant difference between bortezomib plus melphalan and prednisone (VMP) therapy compared to MP therapy in terms of CR, time to progression (TTP), and overall survival (OS), VMP is now recognized as a standard therapy for MM patients aged ≥ 65 years [10]. A retrospective analysis of the VISTA study suggested that higher cumulative doses of bortezomib, reflecting prolonged treatment duration and/or greater dose intensity, could lead to improved OS [11].

The incidence of MM in Korea has increased steadily, and it is the second most common hematologic malignancy in Korea since 2012 [12]. Currently, VMP is the most commonly used regimen for treating patients who are ineligible for transplantation. However, data on the efficacy and safety of VMP therapy in Asian patients with MM are limited.

Thus, we performed a prospective, multicenter, observational study to investigate the clinical effectiveness and safety of VMP treatment for Korean patients with MM.

## RESULTS

### Patient characteristics

In total, 179 patients were enrolled into this observational study at 38 centers in Korea from May 22, 2011 to May 29, 2014, of whom 177, who received at least 1 dose of the study drug, were included in the analyses. The study population comprised 102 (57.6%) men and 75 (42.4%) women, with a median age of 71 years; 39 (22%) patients were aged ≥ 75 years. The most common subtype of MM at the time of diagnosis was immunoglobulin (Ig) G type of MM in 108 (61.0%) patients, followed by IgA type in 43 (24.3%) patients, and IgM type in 2 (1.1%) patients. Overall, 20 (11.3%) patients had light chain disease and 4 (2.3%) had the non-secretory type of MM. According to the International Staging System (ISS), 22 (12.6%) patients were in stage I, 70 (40.0%) were in stage II, and 83 (47.4%) were in stage III (Table 1). In total, 83 (47.4%) patients had a serum β_2_-microglobulin level of > 5.5 mg/L and 129 (73.7%) had glomerular filtration rate < 60 mL/min.

**Table 1:** Patient demographics and baseline characteristics

Category	*N* = 177	
	n	(%)
Sex		
Male	102	(57.6)
Female	75	(42.4)
Age (years)		
Median (range)	71 (48–86)	
≥ 75 years	39	(22.0)
Type of myeloma		
IgG	108	(61.0)
IgA	43	(24.3)
IgM	2	(1.1)
Light chain	20	(11.3)
Non-secretory	4	(2.3)
Stage - Durie–Salmon Staging System		
I	13	(7.3)
II	51	(28.8)
III	113	(63.8)
Stage - International Staging System		
I	22	(12.6)
II	70	(40.0)
III	83	(47.4)
Skeletal lesions		
0	33	(18.6)
1–2	44	(24.9)
≥ 3	80	(45.2)
Unknown	20	(11.3)
ECOG-PS		
0	11	(6.2)
1	104	(58.8)
2	42	(23.7)
3	19	(10.7)
4	1	(0.6)
Serum β_2_-microglobulin (mg/L)		
Median (range)	5.3 (0–35.3)	
< 2.5	17	(9.7)
2.5–5.5	75	(42.9)
> 5.5	83	(47.4)
Albumin (g/dL)		
Median (range)	3.3 (1.0–5.1)	
< 3.5	107	(61.1)
≥ 3.5	68	(38.9)
Creatinine clearance (mL/min)		
Median (range)	43.6 (5.8–106.6)	
< 60	129	(73.7)
≥ 60	46	(26.3)
Comorbidity		
No	40	(22.6)
Yes^b^	137	(77.4)
Allergy	1	(0.6)
Renal impairment	31	(17.5)
Hepatic impairment	5	(2.8)
Others	129	(72.9)
Cytogenetics abnormality category		
Standard risk	121	(68.4)
High risk^a^	24	(13.6)
Deletion 13 by karyotype^b^	17	(9.6)
Deletion 17p by FISH^b^	7	(4.0)
t(4:14) by FISH^b^	6	(3.4)
t(14:16) by FISH^b^	4	(2.3)
Hypodiploidy by karyotype^b^	2	(1.1)
Test not done	32	(18.1)

^a^ High risk cytogenetics: deletion 13 by karyotype, deletion 17p by FISH/karyotype, t(4;14), t(14;16), and hypodiploidy.

^b^ Multiple counting.

FISH, fluorescence *in situ* hybridization.

Cytogenetic test results were collected in 145 patients at the time of diagnosis of MM. The high-risk cytogenetics group included 24 (13.6%) patients, classified on the basis of karyotypic deletion 13 or hypodiploidy or the presence of deletion 17p, translocation (4;14), or translocation (14;16) on interphase florescence *in situ* hybridization.

### Treatment data

At 100% dose intensity, the maximum planned cumulative dose of bortezomib was 67.6 mg/m^2^, including 41.6 mg/m^2^ during cycles 1–4 and 26 mg/m^2^ during cycles 5–9. The maximum planned cumulative dose of melphalan and prednisone was 324 mg/m^2^ and 2160 mg/m^2^, respectively. The median number of cycles delivered was 5 (range, 1–9), and the median cumulative dose of bortezomib was 35.1 mg/m^2^. The dose intensity of bortezomib, which is the median cumulative dose during the initial 4 cycles, was 25.6 mg/m^2^ (median relative dose intensity (RDI), 83.08%), while the median cumulative dose intensity from cycle 5 onwards was 20.0 mg/m^2^ (median RDI, 87.35%). The median cumulative dose of melphalan and prednisone was 144 mg/m^2^ and 1200 mg/m^2^, respectively. Of 177 patients, 58 completed 9 cycles of VMP therapy and 52 completed the 2-year follow-up. Among 119 patients who terminated the therapy before the end of the 9 cycles, the most common reason for termination of therapy was adverse events (AEs) in 26 patients, death in 20 patients, disease progression in 19 patients, and initiation of the next therapy in 17 patients. For 39 patients who were aged ≥ 75 years, the median number of treatment cycles was 3, and the median cumulative dose of bortezomib was 28.8 mg/m^2^.

### Treatment response

**Figure 1:**
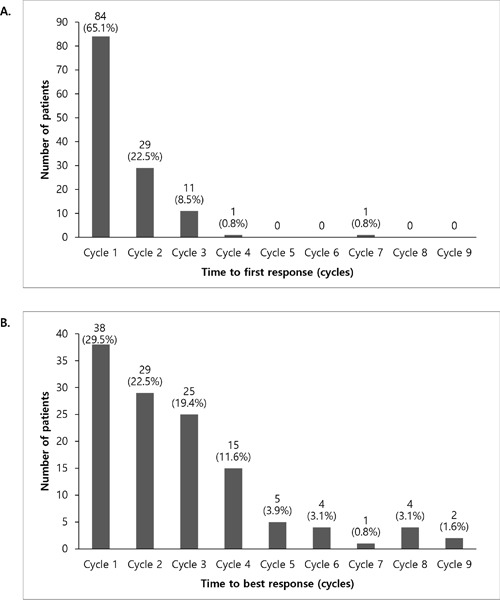
Time to first response **A**. and time to best response **B**.

Response was observed in 129 (72.9%) out of 177 patients (95% confidence interval [CI], 65.7%–79.3%). Details regarding responses to treatment are shown in Table 2. The overall response rate (ORR; stringent CR [sCR], CR, very good partial response [VGPR], and PR) was 72.9%, and the CR rate (sCR and CR) was 20.3%. For 129 patients who responded to therapy, the median time to first response was 1.6 months, mostly in cycles 1 and 2 in 84 (65.1%) and 29 (22.5%) patients, respectively. The median time to best response was 3.4 months, mostly in cycles 1, 2, 3, and 4, each in 38 (29.5%) patients, 29 (22.5%) patients, 25 (19.4%) patients, and 15 (11.6%) patients, respectively (Figure 1). The median duration of response was 17.1 months for the overall population, and 20.6 months for patients with CR.

**Table 2:** Best response to treatment

Category	*N* = 177	%
sCR	3	1.7
CR	33	18.6
VGPR	47	26.6
PR	46	26.0
SD	19	10.7
PD	2	1.1
NE	27	15.3
ORR (≥ PR) (95% CI)	129	72.9 (65.7–79.3)
CRR (sCR + CR) (95% CI)	36	20.3 (14.7–27.0)

CRR, complete response rate; NE, not evaluable; PD, progressive disease; SD, stable disease.

### Survival data

**Figure 2:**
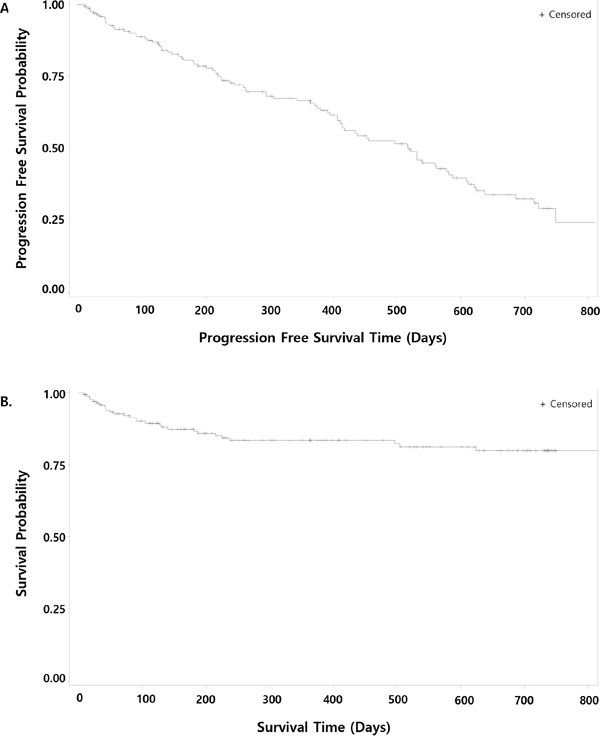
Kaplan–Meier curves for progression-free survival **A**. and overall survival **B**.

The median follow-up duration for 177 patients was 11.9 months. The projected 2-year progression-free survival (PFS) rate was 29.2% (95% CI, 20.4%–38.5%), and the median PFS was 17 months (95% CI, 13.6–19.1 months). The projected 2-year OS rate was 80.0% (95% CI, 71.9%–85.9%) and median OS was not reached (Figure 2). During the safety follow-up period, 26 (14.7%) patients died.

**Figure 3:**
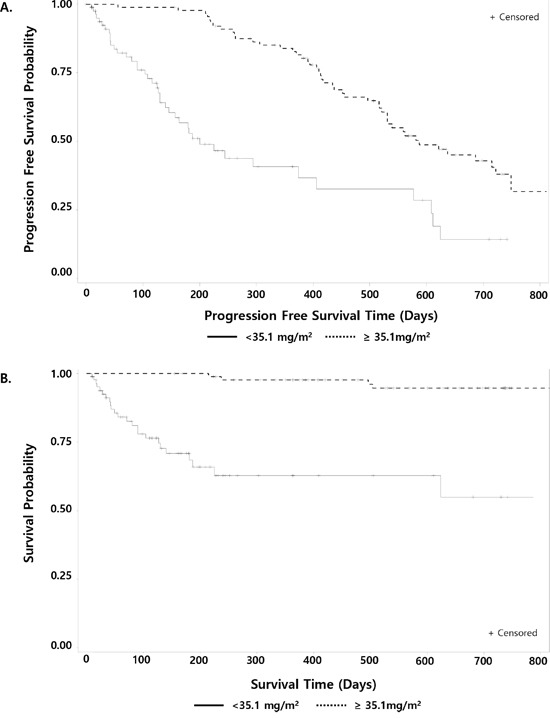
Progression-free survival **A**. and overall survival **B**. by cumulative dose of bortezomib (≥35.1 vs. <35.1 mg/m2).

In univariate analysis for factors affecting 2-year PFS rate, Eastern Cooperative Oncology Group performance status (ECOG-PS) and cumulative dose of bortezomib were significantly associated with PFS, while serum β_2_-microglobulin concentration tended to be associated with PFS (Table 3). Multivariate analysis showed that ECOG-PS < 2 (vs. ≥ 2), β_2_-microglobulin < 5.5 mg/L (vs. ≥ 5.5 mg/L), and cumulative dose of bortezomib ≥ 35.1 mg/m^2^ (vs. < 35.1 mg/m^2^) were independent predictors for longer PFS (Table 4). β_2_-microglobulin < 5.5 mg/L, albumin ≥ 3.5 g/L, and cumulative dose of bortezomib ≥ 35.1 mg/m^2^were independent prognostic factors for OS (Table 4). Patient age ≥ 75 years, impaired renal function, advanced ISS stage, and high-risk cytogenetic abnormalities did not significantly affect the PFS and OS. Figure 3 shows the PFS and OS according to cumulative bortezomib dose (≥ 35.1 vs. < 35.1 mg/m^2^).

**Table 3:** Univariate analysis for progression-free survival and overall survival

Group	*n*	2-year PFS	*p* value^a^	2-year OS	*p* value^a^
Age, years			0.415		0.821
< 75	138	28.6		80.5	
≥ 75	39	31.2		78.6	
Sex			0.520		0.305
Male	102	28.1		76.4	
Female	75	31.2		85.6	
ECOG-PS			< 0.0001		0.005
0, 1	115	37.6		84.9	
≥ 2	62	13.4		70.8	
β_2_-microglobulin, mg/dL^b^			0.065		0.029
< 2.5	17	80.1		87.4	
2.5–5.5	75	23.8		88.8	
> 5.5	83	25.9		70.9	
Albumin, g/dL^b^			0.344		0.063
< 3.5	107	31.4		75.4	
≥ 3.5	68	25.6		86.2	
ISS stage^b^			0.220		0.025
I	22	0.0		88.9	
II	70	35.2		88.2	
III	83	25.9		70.9	
Creatinine clearance, mL/min^b^			0.618		0.459
≥ 60	129	28.6		79.1	
< 60	46	31.7		81.9	
Cytogenetics risk^b^			0.312		0.298
Standard	121	25.9		77.5	
High	24	43.3		90.6	
Cumulative dose of bortezomib			< 0.0001		< 0.0001
< 35.1 mg/m^2^	88	14.3		54.9	
≥ 35.1 mg/m^2^	89	38.0		94.7	

^a^ Log-rank test *p* value.

^b^ Microglobulin values missing in 2 patients, albumin values missing in 2 patients, ISS stage values missing in 2 patients, creatinine clearance values missing in 2 patients, and cytogenetics risk assessment missing in 24 patients.

**Table 4:** Multivariate Cox proportional analysis for progression-free survival and overall survival

Factors	PFS				OS			
	HR	95% HR CI		*p* value^a^	HR	95% HR CI		*p* value^a^
		(Lower,	Upper)			(Lower,	Upper)	
ECOG-PS, ≥ 2	2.236	(1.454,	3.438)	0.0002	-	-		-
β_2_-microglobulin, ≥ 5.5 mg/dL	1.549	(1.004,	2.390)	0.0481	3.624	(1.546,	8.492)	0.003
Cumulative dose of bortezomib ≥ 35.1 mg/m^2^	0.287	(0.181,	0.453)	< 0.0001	0.048	(0.015,	0.153)	< 0.0001
Albumin, ≥ 3.5 g/dL	-	-		-	0.344	(0.142,	0.835)	0.0183

HR, hazard ratio.

^a^ Cox proportional hazards regression model.

Model Fit and Testing Global Ho- Likelihood ratio test: *p* = < 0.0001.

### Adverse events

Overall, 174 (98.3%) patients reported 2,072 AEs, and 154 (87.0%) patients reported 1,095 adverse drug reactions (ADRs). Serious adverse events (SAEs) were reported by 121 (68.4%) patients (277 events). Of these, 29 AEs in 26 (14.7%) patients resulted in death. The most common AE was diarrhea in 71 (40.1%) patients, followed by asthenia in 66 (37.3%) patients, peripheral neuropathy in 60 (33.9%) patients, decreased appetite in 53 (29.9%) patients, and nausea in 50 (28.3%) patients. Major hematologic AEs were cytopenias; grade 3 or 4 neutropenia (14.7%), thrombocytopenia (10.7%), and anemia (3.4%) were frequently observed (Table 5).

**Table 5:** Adverse events

Adverse Events	Total	Grade 3	Grade 4
		*n* (%)	
Any event	174 (98.3)	110 (62.2)	32 (18.1)
Gastrointestinal disorders			
Diarrhea	71 (40.1)	25 (14.1)	1 (0.6)
Nausea	50 (28.3)	2 (1.1)	0 (0.0)
Constipation	48 (27.1)	2 (1.1)	0 (0.0)
Vomiting	31 (17.5)	2 (1.1)	0 (0.0)
Nervous system disorders			
Neuropathy peripheral	60 (33.9)	10 (5.7)	0 (0.0)
Dizziness	30 (17.0)	2 (1.1)	0 (0.0)
Infections and infestations			
Pneumonia	43 (24.3)	16 (9.0)	3 (1.7)
Herpes zoster	27 (15.3)	9 (5.1)	0 (0.0)
Blood and lymphatic system disorders			
Neutropenia	32 (18.1)	19 (10.7)	7 (4.0)
Thrombocytopenia	31 (17.5)	14 (7.9)	5 (2.8)
Anemia	18 (10.2)	6 (3.4)	0 (0.0)
Leukopenia	4 (2.3)	0 (0.0)	1 (0.6)
Other conditions			
Asthenia	66 (37.3)	18 (10.2)	0 (0.0)
Decreased appetite	53 (29.9)	6 (3.4)	0 (0.0)
Pyrexia	43 (24.3)	0 (0.0)	1 (0.6)
Back pain	35 (19.8)	7 (4.0)	0 (0.0)
Fatigue	29 (16.4)	5 (2.8)	0 (0.0)
Dyspnea	28 (15.8)	4 (2.3)	1 (0.6)

There were 380 dose reductions cases in 70 patients (39.5%) during 1–9 cycles; the main cause was AEs in 65 patients (92.8%). There were 4 deaths considered treatment-related: 4 from pneumonia, including 1 from aspiration pneumonia.

## DISCUSSION

Although the superiority of VMP over MP therapy in transplant-ineligible MM patients was demonstrated in the large randomized phase III VISTA study [10], prospective studies supporting the efficacy of VMP therapy in Asian countries are limited. This prospective multicenter study evaluated the efficacy and safety of VMP therapy for Korean patients with MM. Considering the potential gap between efficacy and effectiveness of new treatments in elderly myeloma patients, this population-based observational study is meaningful as it reflects outcomes in clinical practice.

The results were comparable to the overall efficacy results of recently published VMP trials [10, 13–15]. Table 6 summarizes the baseline characteristics and VMP treatment results from recently published studies [11, 14, 15]. The ORR in our study was similar to that from previous VMP studies. However, the PFS of patients in our study was shorter than that of patients in the VISTA study. This discrepancy may be because patients with advanced disease and poor performance were more common in our population. The US community-based phase IIIB UPFRONT study compared three frontline bortezomib-based regimens in transplant-ineligible patients with MM. The median PFS in the UPFRONT study was 17.3 months, similar to our results [14]. This shorter PFS may also reflect the modification of treatment or differences between patients in the community setting. In our study, patients received a median of 5 cycles of VMP treatment compared to 8 cycles in the VISTA trial. The median time to CR was similar to that in the VISTA trial. The CR rate may be lower because the median number of treatment cycles was lower than in other studies. Considering that prolonged treatment might improve the quality of response, early discontinuation of treatment may have negatively affected the outcomes of this study.

**Table 6:** Comparison of results of VMP treatment group among recent studies

	VISTA study	UPFRONT study	Japanese study	Korean study
No. of patients	344	167	87	177
Median age, years	71 (57-90)	72 (68-77)	71 (48-84)	71 (48-86)
≥ 75 years (%)	31	37	27.6	22
ISS stage III (%)	35	36	27.6	47.4
ORR (CR/VGPR/PR) (%)^a^	74	70	69.8	72.9
CR (%)	33	32^c^	19.8	20.3
Time to best response, median (months)^a^	4.2	NA	NA	3.4
Duration of response, median (months)	19.9	19.8	NA	17.1
Number of cycles, median (range)	8	7	4.5	5
PFS, median (months)	24^b^	17.3	NA	17.0
OS, median (months)	56.4	53.1	NA	NA
Neutropenia, any grade (%)	49	23	97	18.1
Thrombocytopenia, any grade (%)	52	18	98	17.5
Peripheral neuropathy, any grade (%)	44	47	67	33.9
Herpes zoster, any grade (%)	13	6	7	15.3
Pneumonia, any grade (%)	16	6	11^d^	24.3

NA, not available.

^a^ Response by International Uniform Response Criteria.

^b^ Time to progression.

^c^ CR + near CR.

^d^ Incidence of lung injury associated with bortezomib.

In a phase I/II study of VMP in Japanese patients, VMP with 1.3 mg/m^2^ of bortezomib showed an ORR of 69.8% and survival outcome was not reported. Similar to our study, the median number of treatment cycles was 4.5 [15]. The incidence of grade 4 neutropenia and thrombocytopenia was 30% and 22%, respectively. Peripheral neuropathy was the most common cause of discontinuation of study treatment. Owing to the limitation of an observational study design, the frequency and severity of AEs in our study and the UPFRONT study seem to be mostly underestimated. However, the incidences of herpes zoster infection and pneumonia were highest in our study. The treatment may have been terminated early due to AEs in both the Japanese and Korean studies.

Post-hoc landmark analysis of the VISTA study suggested that a higher cumulative dose of bortezomib and continued VMP treatment following attainment of CR might result in improved OS [11]. In our subgroup analysis, cumulative dose of bortezomib was also an important factor for favorable outcome. Experts have recommended prompt dose adjustment of novel myeloma drugs according to the patient's condition because continuous treatment until disease progression or intolerability increases the depth of response and extended survival [16]. Early adjustment of treatment according to frailty status and AEs is essential to continue treatment and improve outcome of patients treated with VMP regimen. Subcutaneous injection of bortezomib showed improvements in tolerability of treatment [17]. A less intensive VMP regimen with weekly bortezomib schedule was investigated to prolong treatment duration, and similar outcomes were achieved [16, 18]. Dose reductions based on geriatric assessment may help reduce treatment toxicity and avoid early discontinuation of treatment [19].

Intriguingly, none of the potential prognostic factors such as advanced age, renal impairment, and high-risk cytogenetic profiles were found to predict survival with VMP therapy. This is consistent with the results of previous studies with bortezomib, suggesting that it may overcome some of the poor prognostic impact of these factors [9–11, 13]. Although VMP therapy is a well-established standard treatment for patients with MM who are ineligible for high-dose therapy, it is not clear whether very elderly patients should be treated with VMP, considering the toxicities. In our study, there was no difference in PFS for patients who were aged ≥ 75 years. Based on this, it is suggested that VMP may be an effective treatment option even in very elderly patients. Few studies have been dedicated to patients over the age of 75 years, and currently no studies have been designed based on frailty. Prospective studies are warranted to improve outcomes of these populations. Recently, alkylator-free continuous lenalidomide-dexamethasone (Rd) given until disease progression showed superior PFS and OS to melphalan-prednisone-thalidomide (MPT) in transplant-ineligible patients with myeloma [20]. According to the updated results, continuous Rd also demonstrated benefits over MPT for patients older than 75 years [21]. Currently, both fixed duration of alklyator-combining therapy and continuous alklyator-free regimen are considered as standard treatment for transplant-ineligible MM patients. Further prospective studies may clarify strategies that are more effective for elderly patients.

This is one of the largest multicenter studies to evaluate prospectively the efficacy of VMP therapy in transplant-ineligible MM patients in Asian countries. VMP therapy showed similar results as in previous studies in Korean MM patients. We confirmed that VMP is a feasible and effective front-line treatment for transplant-ineligible older patients with MM in Korea. Continuing therapy with prompt adjustment of treatment according to AEs may be important to improve outcomes of elderly patients.

## MATERIALS AND METHODS

### Ethics statement

Investigation has been conducted in accordance with the ethical standards and according to the Declaration of Helsinki and according to national and international guidelines and has been approved by the authors' institutional review board.

### Patients

The study population included patients with untreated, symptomatic, measurable MM who were not eligible for autologous stem cell transplantation. Symptomatic MM was defined as the presence of intramedullary monoclonal plasma cells ≥ 10% or histologically confirmed plasmacytoma, or presence of monoclonal protein in the serum or urine, or myeloma-related organ impairment. Measurable disease was defined as the presence of quantifiable M protein in serum or urine for secretory MM or confirmed abnormal free light chain ratio for non-secretory MM. The exclusion criteria were asymptomatic MM or monoclonal gammopathy of undetermined significance, previous treatment for MM, severe peripheral neuropathy (grade ≥ 2 according to National Cancer Institute Common Toxicity Criteria for Adverse Events [NCI-CTCAE] version 4.0), pregnancy or breastfeeding, mental illness, or other serious medical conditions.

### Study design and treatments

The patients received VMP treatment guided by approved label which comprised nine 6-week cycles of melphalan (at a dose of 9 mg/m^2^) and prednisone (at a dose of 60 mg/m^2^) on days 1 to 4 in combination with bortezomib (at a dose of 1.3 mg/m^2^) on days 1, 4, 8, 11, 22, 25, 29, and 32 during cycles 1 to 4, and on days 1, 8, 22, and 29 during cycles 5 to 9.

With one cycle consisting of 6 weeks (42 days) from the day when VMP was started, the planned treatment duration was up to 9 cycles, and the follow-up period was 2 years from the day when VMP was started. Considering the observational nature of the study, doses, administration interval, and total treatment cycles could be modified based on the investigator's discretion. After completion of treatment or withdrawal of study drug (due to SAEs or disease progression), patients were followed up for 2 years from the start of VMP treatment. Follow-up was discontinued and considered completed when the next therapy was initiated because of disease progression or death during the 9 cycles of VMP therapy or the follow-up period.

### Study endpoints and assessments

The primary endpoint was the 2-year PFS rate. The secondary endpoints were ORR, CR rate, time to response, and OS. Response and progression were assessed by investigators according to the International Myeloma Working Group uniform criteria [22].

Efficacy and safety were evaluated for all subjects who had received at least one dose of bortezomib. All AEs were reported from the start date of bortezomib administration to 30 days after the last administration date. The severity of the AEs was evaluated according to the NCI-CTCAE version 4.0 (http://ctep.cancer.gov/protocolDevelopment/electronic_applications/ctc.htm).

PFS was defined as the length of time from the day of bortezomib first administration to disease progression, relapse from CR, or death, whichever occurred first, in 2 years. OS was defined as the time between bortezomib first administration and death. Death, regardless of the cause, was considered as an event. Duration of response was defined as the time from the date of first evidence of achievement of at least a minor response until date of disease progression, relapse, or death from any cause.

### Statistical analysis

Demographic and baseline data as well as effectiveness data were summarized using descriptive statistics. Descriptive statistics were presented as continuous variables, which included the mean, standard deviation, median, and range (minimum and maximum), and as categorical variables, which included frequencies and their respective percentages. Time-to-event outcome, including OS, PFS, and duration of response, was analyzed using Kaplan–Meier method.

Differences between groups were compared using the chi-square test or Fisher's exact test for categorical variables and *t*-test for continuous variables. Survival curves between subgroups were compared using the log-rank test for univariate analysis, and multivariate analysis was performed using Cox's proportional hazard model for survival. Factors with *p* values < 0.1 in univariate analyses were examined using multivariate regression models. For the multivariate analyses, a stepwise approach was used. All statistical tests were 2-sided, with significance defined as *p* < 0.05. Analyses were performed using SAS version 9.2.

## Abbreviations

AE: adverse event
CI: confidence interval
CR: complete response
ECOG: Eastern Cooperative Oncology Group
Ig: immunoglobulin
ISS: International Staging System
MM: multiple myeloma
MP: melphalan-prednisone
PFS: progression-free survival
PR: partial response
ORR: overall response rate
OS: overall survival
RDI: relative dose intensity
SAE: serious adverse event
sCR: stringent complete response
VGPR: very good partial response
VMP: bortezomib-melphalan-prednisone.
